# Tracking online searches for gambling activities and operators in the United Kingdom during the COVID-19 pandemic: A Google Trends™ analysis

**DOI:** 10.1556/2006.2023.00055

**Published:** 2023-10-12

**Authors:** Scott Houghton, Frederic Boy, Alexander Bradley, Richard James, Heather Wardle, Simon Dymond

**Affiliations:** 1 School of Psychology, Swansea University, Singleton Campus, Swansea, SA2 8PP, United Kingdom; 2 Department of Psychology, Northumbria University, Newcastle Upon Type, NE1 8ST, United Kingdom; 3 iLab Innovation and Research Centre, School of Management, Swansea University, Bay Campus, Swansea, SA2 8PP, United Kingdom; 4 School of Education and Sociology, University of Portsmouth, Portsmouth, PO1 2HY, United Kingdom; 5 School of Psychology, University of Nottingham, Nottingham, NG7 2RD, United Kingdom; 6 School of Social and Political Sciences, University of Glasgow, Glasgow, G12 8QQ, United Kingdom; 7 Department of Psychology, Reykjavík University, Menntavegur 1, Nauthólsvík, 101 Reykjavík, Iceland

**Keywords:** COVID-19, pandemic, public health, gambling, activities, operators

## Abstract

**Background:**

Whilst some research has explored the impact of COVID-19 on gambling behaviour, little is yet known about online search behaviours for gambling during this period. The current study explored gambling-related online searches before, during and after the outbreak of the COVID-19 pandemic in the UK. We also assessed whether search trends were related to Gambling Commission behavioural data over the same period.

**Methods:**

Google Trends™ search data, covering thirty months from January 2020 to June 2022, for five gambling activities and five gambling operators were downloaded. Graphical displays of the weekly relative search values over this period were then produced to visualise trends in search terms, with key dates in COVID-19 policy and sporting events highlighted. Cross-correlations between seasonally adjusted monthly search data and behavioural indices were conducted.

**Results:**

Sharp increases in internet searches for *poker*, *slots*, and *bingo* were evident during the first lockdown in the UK, with operator searches sharply decreasing over this period. No changes in gambling activity searches were highlighted during subsequent lockdowns, although small increases in operator-based searches were detected. Strong positive correlations were found between search data and industry data for *sports betting* and *poker* but not for *slots*.

**Conclusions:**

Google Trends™ data may act as an indicator of population-level gambling behaviour. Substitution of preferred gambling activities for others may have occurred during the first lockdown when opportunities for sports betting were limited. Further research is needed to assess the effectiveness of internet search data in predicting gambling-related harm.

## Introduction

In the United Kingdom (UK), politicians, public health officials, and regulators have raised concerns about the impact of the COVID-19 pandemic on gambling behaviours (All Party Parliamentary Group on Gambling Harms, 2020; Gambling Commission, 2020). It was feared that the introduction of COVID-19 lockdown restrictions in March 2020 may exacerbate gambling harms through changing financial/employment circumstances, increased psychosocial isolation, stress, and anxiety. At that time, all offline (i.e., land-based) gambling venues were closed, and all professional sports (including horse racing) were suspended until June 2020. Since then, there has been a gradual return of professional sports, while gambling venues reopened with social distancing and face-covering requirements in May 2021 after a third national lockdown. In August 2021, all restrictions were lifted.

These restrictions to offline gambling for large periods in both 2020 and 2021 raised concerns about people switching to online gambling, primarily, which is often seen as a riskier form of gambling (Papineau et al., 2018). The impact of COVID-19 on gambling has however been more nuanced than expected, although the research picture remains incomplete as the effects of the pandemic on population mental well-being continue to be felt (Brodeur, Clark, Fleche, & Powdthavee, 2021). Increased vulnerability to gambling harms has however been shown to increase in some groups who changed their gambling behaviours during lockdown (e.g., regular sports bettors), such as through increased frequency of gambling or gambling on different activities (Wardle et al., 2021). Those groups that increased their gambling during lockdown(s) tended to be younger, male, and with gambling severity scores indicating potential trajectories of at-risk gambling behaviours (Hodgins & Stevens, 2021).

During the first national lockdown, paid-for advertising in the UK dropped 38.5% from the same time period in the previous year (Critchlow, Hunt, Wardle, & Stead, 2022). However, a 49.3% increase in spend upon the previous year was identified for the second lockdown and a 5.3% increase from the previous year was observed for the third lockdown (see Supplementary Materials for details of lockdown dates and restrictions). Whilst reduced spending during the first lockdown aligns with research suggesting overall reductions in gambling behaviour during that period (Sharman, Roberts, Bowden-Jones, & Strang, 2021; Wardle et al., 2021), increased advertising during subsequent lockdowns may have promoted more opportunities to gamble online and therefore potentially exacerbated harms. Whilst the overall gross gambling yield (GGY) of the UK gambling industry decreased from £14.25 billion for the 12 months between April 2019 and March 2020 (Gambling Commission, 2022b) to £12.68 billion in the following year, there was an 16% increase in online GGY. Therefore, this decrease was largely due to declines across all offline sectors (Gambling Commission, 2020, 2022b). In this way, it appears that the methods used by people to gamble may have changed because of the onset of the COVID-19 pandemic.

It is important to note that gambling industry revenues provide an incomplete picture of gambling behaviour and most of the studies on the impact of COVID-19 upon gambling rely on retrospective self-report (Sachdeva, Sharma, & Sarangi, 2022), which can be flawed and are susceptible to memory biases (Auer, Malischnig, & Griffiths, 2014; Braverman, Tom, & Shaffer, 2014) and self-presentation biases (Kopcha & Sullivan, 2007). There is, therefore, scope for novel approaches that use objective, real-time Internet datamining to reveal changes in gambling behaviour due to COVID-19 (Springer, Zieger, & Strzelecki, 2021). Google Trends™ is a publicly available information resource from Google Inc. that captures the volume of real-time Internet searches, for given search terms or phrases (Arora, McKee, & Stuckler, 2019). To date, Google Trends™ data have been used to examine the impact of COVID-19 lockdowns on well-being concerns (Brodeur et al., 2021; Knipe, Gunnell, Evans, John, & Fancourt, 2021; Roy, Deb, & Chakarwarti, 2023) and in disease surveillance (Mavragani & Gkillas, 2020; Mavragani & Ochoa, 2019; Mavragani, Ochoa, & Tsagarakis, 2018), amongst other public health related topics. One recent study investigated whether Google Trends™ data for wellbeing-related searches predicted levels of self-reported mental well-being (Knipe et al., 2021). The authors found that, for example, searches for “loneliness” predicted self-reported loneliness from cross-sectional data covering the same period. The combination of Google Trends™ data with objective (albeit self-report based) indices therefore affords researchers and policymakers with a promising new means of detecting population-wide public health trends. Additionally, the triangulation of industry and search data will help address the previously noted limitation of retrospective self-report data when assessing the impact of COVID-19 upon gambling behaviour.

To our knowledge, no published study has reported Google Trends™ data on gambling-related searches. According to journalistic reports, online Google searches for the term "casino" increased in 25 UK cities during the first national lockdown (BBC, 2020). In its submission to the House of Commons Digital, Culture, Media, and Sport Select Committee in June 2020, the charity (GambleAware, 2020) noted a 193% increase in searches for online betting and virtual events in the two weeks post-lockdown in March 2020, with an initial spike in searches for ‘poker’ and ‘sports betting’ followed by a return to pre-lockdown levels. The submission also noted a steady increase in searches relating to ‘online gambling’ as the resumption of the Premier League football competition approached. Such increases in searches for online gambling may be the result of substitution from in-person gambling to online gambling (Xuereb, Kim, Clark, & Wohl, 2021), given the restrictions on access to land-based gambling venues during the first national UK lockdown. Since then, little is known about the impact of the COVID-19 pandemic on Internet searches for gambling-related terms. Additionally, to capitalise on the growing interest in search data from policymakers, there is a need for a more systematic approach to fully investigate such trends across a range of gambling activities and operators.

The present study sought, for the first time, to formally investigate Google Trends™ data to answer the research question: “how were gambling-related topics searched for before, during, and since the introduction of COVID-19 restrictions in the UK?” We aimed to identify how gambling-related search trends may or may not have been impacted by significant events in the gambling calendar, such as the suspension of professional sports and the impact of increased gambling advertising during lockdown on the online profile of popular UK gambling operators. Finally, we also aimed to explore whether the Google Trends™ data were related to population-level monthly data reported by operators on gross gambling yield, number of active players, and total number of bets placed.

## Methods

### Data source

We analysed Google Trends™ data, which is a publicly available data service provided by Google Inc. allowing internet users to access time-series data on keyword-based internet searches freely. Whilst there are other search engines, Google had 91.74% of the market share across devices in the 12 months from July 2021 (Statcounter, 2022) and therefore represents the majority of online search behaviours. Google Trends™ provides access to a single standardised metric: the Relative Search Volume (RSV) for a specific search term of interest, or a combination of search terms. This metric is standardised relative to all other search terms within a specified location and within a specific time-period. As such, the total number of searches for a specific term are divided by the total number of searches within a particular geographic region over the specified time-period of the search. Google then rescale the resulting estimates to assign an RSV in the range of 0–100 based on the search's popularity compared to all searches on all topics. Therefore, a higher RSV indicates a higher search volume for that search term within the temporal and geographical parameters set. The search volume algorithm assigns a zero-value to periods with minimal numbers of searches.

### Search strategy

Searches were carried out on July 5th 2022. All seven-day RSV datasets were extracted for the thirty months between January 1st 2020 (01/01/2020) and June 30th 2022 (30/06/2022). These dates were chosen to incorporate the time period from the initial outbreak of COVID-19 through to the date of the data extraction. All UK-wide searches from all Google query categories were included.

We first selected four specific gambling activity search terms based on their inclusion within online operator data reports issued by the Gambling Commission (Gambling Commission, 2020; 2022b) throughout the COVID-19 pandemic (*slots, bingo, poker, sports betting*) and the general topic search term, ‘*gambling*’. The Gambling Commission is the UK gambling regulator, with operators required to provide regulatory returns for each license activity they hold, and therefore provide an objective source of industry data.

We selected search terms based on five popular gambling industry operators (Headline Casinos, 2020) – Bet365, Ladbrokes, Paddy Power, Sky Betting and Gaming, and William Hill - and retrieved search data for these terms. These gambling operators account for a significant proportion of the estimated £5.3 billion GGY for the UK online gambling sector in 2019 and are viewed positively by players (YouGov, 2022). The inclusion of operator-specific search terms sought to identify other ways in which individuals may search for sports betting and gambling opportunities, given the high level of gambling integration in sport (Sharman, 2022) and subsequent high brand recognition among people who gamble in the UK (Djohari, Weston, Cassidy, Wemyss, & Thomas, 2019).

### Data analysis

#### Time trends

**Fig. 1.**
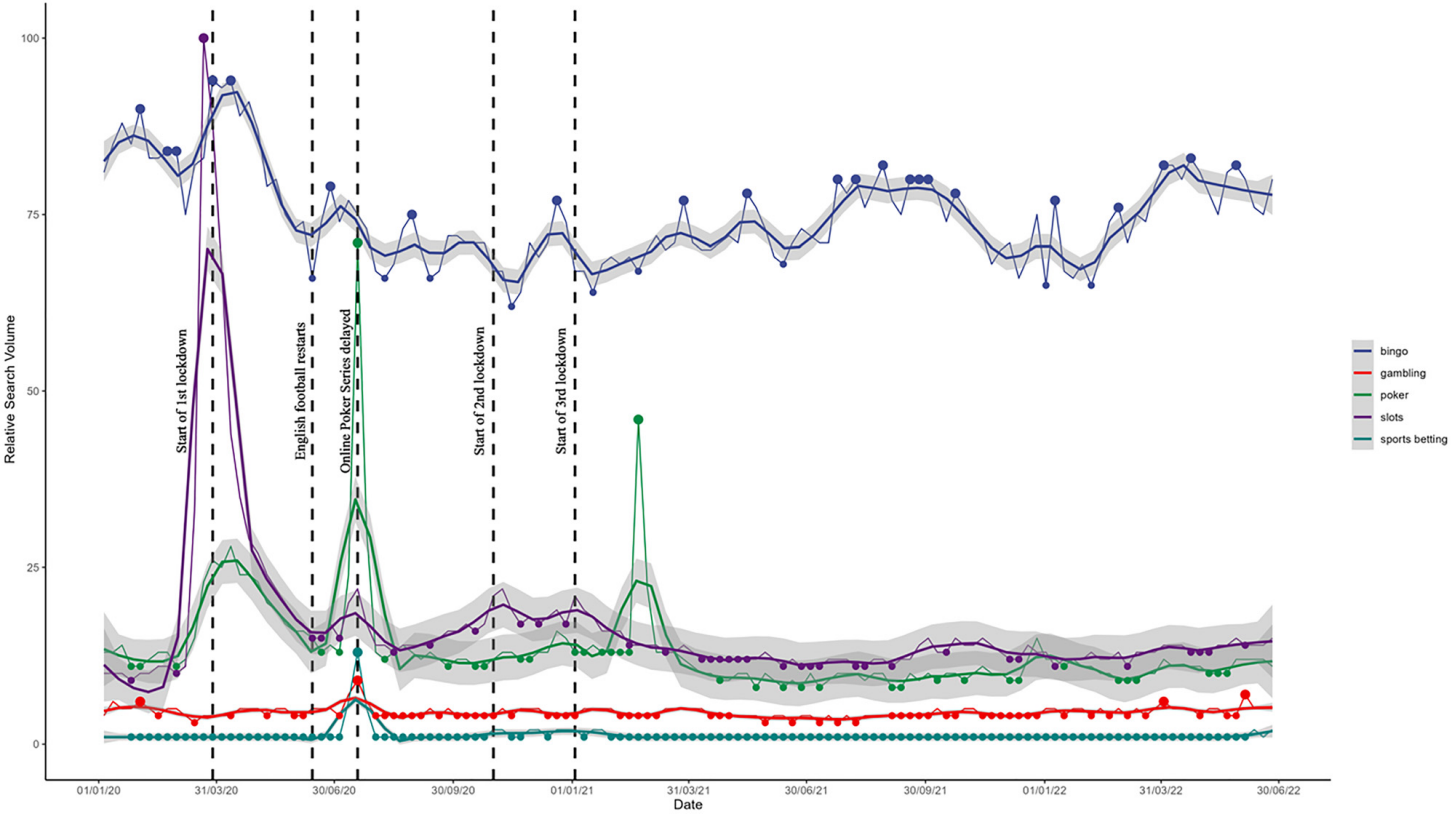
Google searches for gambling activities every week between 01/01/2020 and 30/06/2022 *Note*. 1^st^ national lockdown (26/03/20 to 23/06/20). 2^nd^ national lockdown (05/11/20 to (02/12/20). 3^rd^ national lockdown (06/01/21 to 19/07/21).

**Fig. 2.**
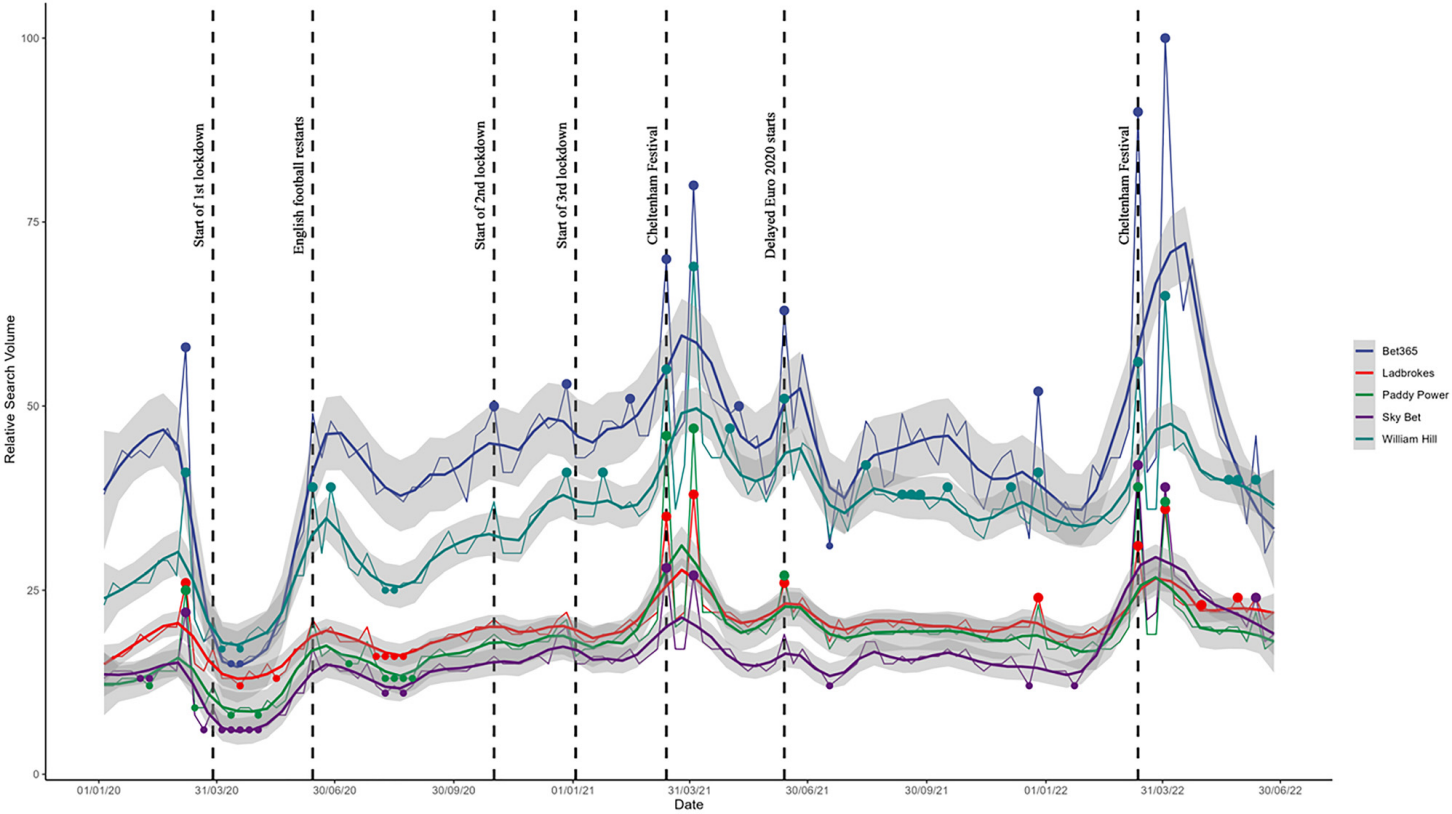
Google searches for gambling operators every week between 01/01/2020 and 30/06/2022 *Note*. 1^st^ national lockdown (26/03/20 to 23/06/20). 2^nd^ national lockdown (05/11/20 to (02/12/20). 3^rd^ national lockdown (06/01/21 to 19/07/21).

We produced graphical displays of average RSVs timeseries for gambling activities and gambling operator search terms, arranged weekly between 01/01/2020 and 30/06/2022 (see Figs 1 and 2). Important dates in the implementation of the UK's COVID-19 lockdown restrictions policies and significant events in the gambling landscape are highlighted. Similar graphs were also created for both sets of search terms for the 3 years previous (01/01/2017 to 31/12/2019) to understand the seasonality of search terms (see Supplementary Materials).

#### Cross-correlations with operator data

To investigate whether Google Trends™ data could act as a proxy measure for gambling behaviour over time, a series of cross-correlations were carried out between monthly RSVs and monthly operator data from the regulator, the Gambling Commission (Gambling Commission, 2022a). The data covered online gambling over a 25-month period from March 2020 to March 2022. It included data from the biggest operators in the UK and captured around 80% of the online gambling market. Data included within the analysis was the number of active players per month, the total number of bets placed per month, and gross gambling yield per month for each of three activities: *sports betting*, *slots*, and *poker*. Monthly RSV timeseries were created by averaging the weekly RSVs for each month. An average betting industry RSV was then calculated by averaging the monthly RSVs across all five operators. Given the nature of time series data and the potential issues this can cause with autocorrelation (Bernal, Cummins, & Gasparrini, 2017), an autocorrelation function (ACF) plot was created for each of the correlations. No significant issues were identified from this. However, there did appear to be an effect of seasonality within the data; therefore, the time-series analysis for each of the search terms and the industry data were decomposed, allowing for seasonality to be subtracted from each period. Cross correlations were then conducted on the adjusted time-series between variable pairs of interest (i.e., poker search terms with poker industry data).

#### Sensitivity analysis

We repeated the searches one month after the original searches (05/08/22) and used Pearson's Correlation Coefficient to determine the sensitivity of the original dataset with the validation datasets.

All analyses were conducted in R (version 4) and all packages, scripts and datasets are available via OSF (https://osf.io/894tf/?view_only=0647f42e53ca47b1af72caeea8b4eb90).

### Ethics

The Research Ethics Committee at Swansea University granted this study an exemption as it constituted a secondary analysis of publicly available data.

## Results

### Sensitivity analysis

There were highly significant correlations between the RSVs within the initial search and the subsequent search one month later for both betting activities (*r* = 0.99, *p* < 0.001) and operators (*r* = 0.99, *p* < 0.001), indicating stability in the RSVs regardless of when the database was searched.

### Time trend findings

#### Gambling activities

Figure 1 shows the weekly RSVs for the four gambling activities and the general search term of *gambling* throughout the search period. *Bingo* was the most popular search term, whilst searches for *gambling* and *sports betting* remained relatively low throughout. There were sharp increases in *bingo*, *poker*, and *slots* searche*s* when the UK entered its first lockdown in March 2020. Search volumes for each of these activities then decreased as restrictions started to be lifted and the English football leagues resumed in June 2020. However, *bingo* search volume decreased to a lower value than pre-lockdown, and *slots* search volume remained higher than it was pre-lockdown. Similar patterns were not observed for either of the other national lockdowns, with RSVs remaining relatively consistent when each lockdown was implemented in November 2020 and January 2021, respectively. There were also spikes in *poker* searches in July 2020 and March 2021, corresponding with the dates of international online poker tournaments.

#### Gambling operators

Figure 2 shows the weekly RSVs for the five operators. There was a clear decrease in search volume for each operator immediately prior to the implementation of the first UK lockdown – falling in line with the suspension of English football (i.e., Premier League and the English Football Leagues). RSVs for each of the operators then returned to back to their original levels as the English football season restarted in June 2020. There were smaller increases in RSVs for the two most popular operators (Bet365 and Paddy Power) at the beginning of both the second and third national lockdowns; however, similar patterns were not observed for the other three operators. There were seasonal increases in RSVs for all five during the main British horse racing season in both 2021 and 2022 (as indicated by the Cheltenham Festival of Racing in March). Finally, there were also smaller increases in RSVs for all operators, except Sky Bet, at the start of the delayed UEFA European Football Championships in June 2021.

### Correlations with operator data

The industry average RSVs were strongly positively correlated both with the number of active betting players and the number of bets place, whilst also being highly correlated with betting GGY (Table 1). Similarly, there were strong positive correlations between *poker* RSVs and each of the industry measures of poker behaviour: GGY, number of active players, and bets placed. Strong negative correlations were observed between betting the RSVs for *slots*-based searches and slots GGY, *slots* RSVs, and the number of active slots players and slots bets placed. In terms of the cross-correlations, these correlations were strongest at 0 month lag, and remained significant with a ± 1 month lag. The exception to this was the poker cross-correlations, which were significant with a longer positive lag.

**Table 1.** Cross-correlation values of search terms with relevant industry data

	−3 months	−2 months	−1 month	0 month	1 month	2 months	3 months
**Sports Betting**							
*Industry Average RSV and Sports Betting GGY*	−0.16	0.01	0.39	0.55*	0.42*	0.23	0.14
*Industry Average RSV and Active Sports Betting Players*	0.29	0.43*	0.74**	0.95**	0.63*	0.28	0.14
*Industry Average RSV and Sports Bets Placed*	−0.07	0.12	0.60*	0.93**	0.77**	0.35	0.20
**Poker**							
*Poker RSV and Poker GGY*	0.15	0.22	0.48*	0.74**	0.66**	0.62**	0.53*
*Poker RSV and Active Poker Players*	0.17	0.24	0.48*	0.77**	0.67*	0.58*	0.54*
*Poker RSV and Poker Bets Placed*	0.15	0.22	0.49**	0.76**	0.63**	0.57**	0.52**
**Slots**							
*Slots RSV and Slots GGY*	−0.40	−0.37	−0.58*	−0.59*	−0.41	−0.27	−0.24
*Slots RSV and Active Slots Players*	−0.59*	−0.65**	−0.71*	−0.74**	−0.54*	−0.27	−0.17
*Slots RSV and Slots Bets Placed*	−0.42	−0.40	−0.49*	−0.72**	−0.67*	−0.34	−0.22

*Note*: Cross-correlations from −3 months lag to 3 months lag, with the strongest correlation highlighted in bold. * = *p* < 0.05, ** = *p* < 0.001

## Discussion

The current study investigated Google Trends™ search data for gambling-related topics before, during, and since the introduction of COVID-19 restrictions in the UK and examined potential associations with gambling operator data over the same period. We found that searches for each operator decreased during the initial lockdown, whilst searches for *bingo*, *poker*, and *slots* sharply increased. Smaller increases were observed at the start of the second and third lockdowns for gambling operator searches, yet no major changes were observed for activity-based searches from the periods preceding the lockdowns. Operator search data and industry data for sports betting were strongly positively correlated, whilst comparable search data for poker were also strongly associated with our industry measures. Specifically, the number of active players, the total number of bets placed, and gross gambling yield per month were strongly correlated with searches for sports betting and poker. Of the remaining industry data measures, we also noted that search data for *slots* was negatively correlated with operator data for slots gambling.

Findings from this study provide an initial exploration of Google Trends™ search data for gambling throughout the pandemic, building upon existing literature of the impact of lockdowns on searches for a range of different public health topics (Brodeur et al., 2021; Knipe et al., 2021; Mavragani & Gkillas, 2020). This highlighted a shift in search behaviour towards specific gambling activities, such as *bingo*, *slots*, and *poker*, during the first lockdown and a shift away from operator searches. A potential explanation of this trend may be that consumers began searching for online equivalents of their offline gambling activities, given their unavailability offline during the first lockdown. This may particularly be the case for *bingo*, given its popularity as an offline gambling activity (Gambling Commission, 2022b). However, the increase in RSVs for *poker* may be better explained by it providing an opportunity for online social interaction during lockdown, given that it is not as popular offline (Gambling Commission, 2019). It is also likely, given the low relative search volume for the terms *sports betting* and *gambling* and that gambling operator names are primarily known for sports betting, that searches for those operators are, in fact, reflective of searches for sports betting in general. This may explain why searches for *sports betting* and *gambling* did not increase in line with operator-specific searches because it is likely that people use operator-specific search terms when seeking opportunities to gamble on sporting events. Such changes in online search behaviour can therefore also be explained by the suspension of major sporting competitions within the UK that coincided with the first lockdown. This limited the opportunities for people to bet on sports and, therefore may have led to them seeking out alternative gambling activities, known as substitution behaviours (Georgiadou et al., 2022; Xuereb et al., 2021). Supporting this, research highlighted that rates of online gambling, including poker, casino games and bingo, increased during the first lockdown (Emond, Nairn, Collard, & Hollén, 2022). Additionally, a study of regular sports bettors found that many either started betting on new activities during lockdown or increased their betting frequency of other activities during lockdown (Wardle et al., 2021). Therefore, when considered with these observations, the current findings provide further evidence of a change in gambling preferences during the first lockdown.

Whilst much research has focussed on the impact of the first national lockdown on gambling behaviour (Hodgins & Stevens, 2021; Wardle et al., 2021), relatively little research has investigated the two subsequent lockdowns. As such, the current study provides an initial exploration into one aspect of how individuals were searching for gambling-related content on Google over this period. This highlighted a smaller spike in searches for operators, but no difference in activity searches, during both lockdowns. The difference in search behaviour compared to the first lockdown could be explained by the fact there was no suspension of sport during subsequent lockdowns. These lockdowns may therefore have provided an environment for many bettors that encouraged gambling behaviour, with no change in the availability of sports betting. This is particularly relevant given that sports betting accounted for around a third of the gambling market share over the past two years (Gambling Commission, 2022b). Additionally, recent advertising spend research highlighted a 103% increase in advertising spend during the second lockdown (Critchlow et al., 2022) that may also have contributed to the increased search volumes for sports betting during this period. Despite this increased availability of gambling, it is possible that searches for other specific gambling activities (e.g. *bingo, poker* and *slots*) did not increase during subsequent lockdowns as customers may have identified preferred operators earlier with the onset of the first lockdown.

The current study also supports the idea that Google Trends™ data may function as a population-level indicator of some gambling (i.e., poker and sports betting) behaviour. Previous research in other domains has found that Google searches for mental health terms did not positively predict self-reported symptoms of mental health problems but searches for loneliness predicted self-reported loneliness over the same period (Knipe et al., 2021). We found that searches for gambling operators and *poker* correlated with industry data for *sports betting* and *poker*, respectively. However, we also found that searches for *slots* and industry data for slots were negatively correlated. A potential explanation for this negative correlation is the popularity of free-to-play slots games online (Kim & King, 2020) and the fact that the use of social casino games increased over the pandemic (Xuereb et al., 2021) – both of which would not be reflected in the operator data. It is also possible that customers searched for and found their preferred operator during the initial lockdown and had less incentive to switch operators or activities during subsequent lockdowns. In the absence of objective or self-report data, this account must however remain speculative. Overall, then, it may be the case that online search data is only useful in predicting the scale of certain gambling behaviours at the population level, rather than the harm caused by such behaviour. In this way, Google searches for *sports betting* and *poker* may be useful indicators for the volume of gambling undertaken on these products (e.g., number of active players, new accounts opened, etc.) but these metrics are not necessarily indicators of harm. While there is evidence that total consumption is related to gambling harms (Rossow, 2019), future research should assess whether gambling search data can predict a wider range of (self-reported) gambling activities and track potential harms.

A major strength of the current study is that it is the first formal investigation using analysis of Google Trends™ data as a research tool for understanding changes in gambling preferences and activities. This allowed us the opportunity to explore how one element of online behaviour relating to gambling was impacted throughout the course of the COVID-19 pandemic. The pandemic provided a unique gambling landscape whereby certain gambling activities were limited in availability for certain periods and individuals had limited options for social interactions or leisure activities. Periods of lockdown also resulted in people spending a lot of time at home, which is where most online gambling occurs (Gambling Commission, 2021). Additionally, a sensitivity analysis was carried out on the downloaded Google search data. Previous research has highlighted potential inconsistency of search data obtained from Google (Rovetta, 2021). However, the sensitivity analysis demonstrated the reliability of the datasets analysed in this study as there were near-perfect correlations between the data sets downloaded a month apart. Finally, ACF plots were run to ensure no confounds were identified with autocorrelation given the time-series nature of the data (Bernal et al., 2017).

Despite these strengths, a potential limitation of the current study is that the operator data provided by the regulator was only based upon 80% of the online gambling market and therefore may not be a complete reflection of gambling behaviour over this period. However, given these data still covered all major UK operators, it is not anticipated that this would have a major impact upon the relationships observed. A further potential limitation is that we did not account for other methods of searching for gambling opportunities online. For example, searching for gambling apps on smartphones was not detected here and nor was how individuals may access applications that they already have downloaded. This is particularly relevant given that smartphones and other mobile devices are the most popular method of accessing online gambling, particularly among younger generations (Gambling Commission, 2021). Despite this, clear trends emerged within the search data that could be tracked back to key periods throughout the period investigated. Therefore, while caution must be taken in that Google Trends™ data is not reflective of all online search data, it still has a practical application in identifying consistent patterns in online search behaviour.

To build upon the findings of the current study, future research should explore how gambling searches were impacted in different countries in relation to their COVID-19 restrictions and subsequent gambling policy implementations. For example, deposit limits were introduced in Belgium and advertising restrictions were enforced in Spain (Brodeur et al., 2021). Therefore, exploring how such restrictions impacted search behaviour may offer valuable insight into how such approaches to regulation impacted levels of engagement with gambling content online. Additionally, the predicative nature of online search data should be further explored. Whilst the current study showed that monthly RSVs for certain gambling activities showed strong correlations with monthly industry data, it is not yet clear whether such relationships would persist for shorter periods, for example, weekly or daily data. It would also be helpful to evaluate how well search data relates to data on gambling harm or help-seeking behaviours.

## Conclusion

To conclude, the current study provides evidence of a shift in gambling searches during the first UK lockdown, with less severe shifts in behaviour during the subsequent lockdowns. Search data was very strongly correlated with commercial behavioural data for sports betting and poker but not for slots gambling, suggesting search data may act as a potential indicator of changes in population level gambling behaviour for certain gambling activities but not others. Our findings provide an early indication of the public health utility of search history behaviour to track gambling trends. This approach can be utilised by researchers and policy makers to easily and continuously monitor gambling-related searches, allowing for the identification of periods with increased gambling activity. These period of increased activity could be used to launch specific public health messaging campaigns advertising gambling support lines, NHS gambling clinics and self-care gambling tips. Overall, further research is needed to assess the utility of search data in tracking population level gambling harms across cultures and public-health scenarios.

## Funding sources

The work described here was supported by an award from the British Academy/Leverhulme Small Research Grants Scheme (SG2122\211340) to Simon Dymond and Heather Wardle. The open access publishing fees for this article were paid by the Gambling Research Exchange Ontario (GREO). The authors would like to thank both British Academy/Leverhulme and GREO for their financial support that allowed this worked to be conducted and published.

## Authors' contribution

All authors were involved in the development of the study. Scott Houghton was the main data analyst and lead author for the project. All authors contributed towards writing the manuscript and all authors read and approved the final version of the manuscript.

## Conflict of interest

Both Scott Houghton and Simon Dymond either previously received or are currently in receipt of funding from GambleAware, who receive voluntary donations from gambling operators.

Richard James is currently supported by research grants from the Academic Forum for the Study of Gambling (AFSG). The funds for this research were received from regulatory settlements made by gambling operators. Simon Dymond is currently a member of the Executive Committee of the AFSG. Richard James and Frederic Boy were previously investigators on projects funded by the International Center for Responsible Gaming, which is a charitable organisation funded by donations from the US gambling industry. Simon Dymond is currently an investigator on a project funded by the Gambling Commission's Socially Responsible Purposes Fund, which is funded through regulatory settlements made by gambling operators.

In the last three years, Heather Wardle and Simon Dymond have worked on a project funded by GambleAware, looking at gambling and suicidality.

In the past five years Heather Wardle discloses grant funding for gambling-related projects from the National Institute for Health Research, Economic and Social Research Council, Wellcome Trust, Office of Health Improvements and Disparities/Public Health England, Gambling Commission (including from regulatory settlements); Gambling Research Exchange Ontario, Greater London Authority, Greater Manchester Combined Authority and the Department for Culture Media and Sport. In 2018/19 she was funded by GambleAware for a project on gambling and suicide. She has been paid consultancy fees by the Institute of Public Health, Ireland and the National Institute for Economic and Social Research. Between 2015 and 2020, she was Deputy Chair of the Advisory Board for Safer Gambling, remunerated by the Gambling Commission. She is a member of the WHO Panel on gambling. She was paid as an expert witness on gambling by Lambeth and Middlesborough Borough Councils. She received payment for delivery of a webinar by McGill University. She provided unpaid advice on research to GamCare. She has received funding for travel from the Turkish Green Crescent Society. Gambling Regulators European Forum and Alberta Gambling Research Institute. She runs a research consultancy practice for public and third sector bodies – she has never provided consultancy services to the gambling industry.

## Supplementary data

The datasets generated during and/or analysed during the current study are available via OSF at https://osf.io/894tf/.

## Abbreviations

ACF: Autocorrelation Function
BBC: British Broadcasting Corporation
GGY: Gross Gambling Yield
RSV: Relative Search Volume
UK: United Kingdom
